# Impudence

**Published:** 1876-06

**Authors:** 


Impudence.—It surely is a mild expression to characterize as impudent the application of the so-called National Surgical Institute to the General Congress for money, incorporation, and various other benefits and privileges. The establishment, we believe, was to be located in the District of Columbia. The petitioners seem to be gathered from those concerned in the several institutions of like name, scattered through the country—this city being blessed with one of them.

We feel no uneasiness as to the result of their little scheme. Congress is too old to be so taken in, and is too much engaged in important and legitimate work to bestow time on this attempted fraud. However, several prominent medical organizations have forwarded, and very properly, their protests against the granting of the petition. We append here the action of the Atlanta Academy of Medicine at a recent meeting.

Atlanta, Ga., May 30th, 1876.

Whereas, Certain persons are endeavoring to secure the passage of a bill by the Congress of the United States entitled "A bill to incorporate the National Surgical Institute of the District of Columbia;"

And Whereas, We believe that the conferring of a charter upon this organization by Congress will be subversive of the best interests of society, inimical to the welfare of the regularly organized colleges of the United States, derogatory to the dignity of the medical profession, and as favoring the practice of charlatanism, to the injury of the people and at the sacrifice of every principle of justice and right, whereby the ignorant and unwary may become the victims of incompetence, avarice, and cupidity:

*Resolved*, That the Atlanta Academy of Medicine do earnestly protest against said bill.

*Resolved*, That our Representatives in Congress be requested to use their influence to defeat this gigantic scheme.

*Resolved*, That a copy of these resolutions, duly signed by the President and Secretary, be forwarded to the President of the Medical Society of the District of Columbia, and to each member of Congress from Georgia.

J. G. Westmoreland, M.D., Vice-Pres't.
T. M. McIntosh, M.D., Secretary.

Since writing the above, we notice that the Senate committee have reported adversely on the petition. So it dies.

